# Potential Biomarkers of Acute Ischemic Stroke Etiology Revealed by Mass Spectrometry-Based Proteomic Characterization of Formalin-Fixed Paraffin-Embedded Blood Clots

**DOI:** 10.3389/fneur.2022.854846

**Published:** 2022-04-19

**Authors:** Rosanna Rossi, Oana Madalina Mereuta, Mariel Barbachan e Silva, Sara Molina Gil, Andrew Douglas, Abhay Pandit, Michael Gilvarry, Ray McCarthy, Shane O'Connell, Ciara Tierney, Klearchos Psychogios, Georgios Tsivgoulis, István Szikora, Turgut Tatlisumak, Alexandros Rentzos, John Thornton, Pilib Ó Broin, Karen M. Doyle

**Affiliations:** ^1^Department of Physiology and Galway Neuroscience Centre, School of Medicine, National University of Ireland, Galway, Ireland; ^2^CÚRAM–SFI Research Centre for Medical Devices, National University of Ireland Galway, Galway, Ireland; ^3^School of Mathematical and Statistical Sciences, National University of Ireland Galway, Galway, Ireland; ^4^Cerenovus, Galway, Ireland; ^5^Metropolitan Hospital, Stroke Unit, Piraeus, Greece; ^6^Second Department of Neurology, National and Kapodistrian University of Athens, “Attikon” University Hospital, Athens, Greece; ^7^Department of Neurointerventions, National Institute of Clinical Neurosciences, Budapest, Hungary; ^8^Department of Neurology, Sahlgrenska University Hospital, Gothenburg, Sweden; ^9^Department of Clinical Neuroscience, Institute of Neuroscience and Physiology, Sahlgrenska Academy at University of Gothenburg, Gothenburg, Sweden; ^10^Department of Interventional and Diagnostic Neuroradiology, Sahlgrenska University Hospital, University of Gothenburg, Gothenburg, Sweden; ^11^Department of Radiology, Royal College of Surgeons in Ireland, Beaumont Hospital, Dublin, Ireland

**Keywords:** biomarkers stroke etiology, stroke biomarkers, mass spectrometry proteomics, FFPE proteomics, thrombus proteome

## Abstract

**Background and Aims:**

Besides the crucial role in the treatment of acute ischemic stroke (AIS), mechanical thrombectomy represents a unique opportunity for researchers to study the retrieved clots, with the possibility of unveiling biological patterns linked to stroke pathophysiology and etiology. We aimed to develop a shotgun proteomic approach to study and compare the proteome of formalin-fixed paraffin-embedded (FFPE) cardioembolic and large artery atherosclerotic (LAA) clots.

**Methods:**

We used 16 cardioembolic and 15 LAA FFPE thrombi from 31 AIS patients. The thrombus proteome was analyzed by label-free quantitative liquid chromatography-tandem mass spectrometry (LC-MS/MS). MaxQuant v1.5.2.8 and Perseus v.1.6.15.0 were used for bioinformatics analysis. Protein classes were identified using the PANTHER database and the STRING database was used to predict protein interactions.

**Results:**

We identified 1,581 protein groups as part of the AIS thrombus proteome. Fourteen significantly differentially abundant proteins across the two etiologies were identified. Four proteins involved in the ubiquitin-proteasome pathway, blood coagulation or plasminogen activating cascade were identified as significantly abundant in LAA clots. Ten proteins involved in the ubiquitin proteasome-pathway, cytoskeletal remodeling of platelets, platelet adhesion or blood coagulation were identified as significantly abundant in cardioembolic clots.

**Conclusion:**

Our results outlined a set of 14 proteins for a proof-of-principle characterization of cardioembolic and LAA FFPE clots, advancing the proteome profile of AIS human thrombi and understanding the pathophysiology of ischemic stroke.

## Introduction

Stroke is a leading cause of mortality and disability worldwide, with 11.9 million stroke accidents recorded in 2017, 6.2 million of which were fatal (1). The majority of strokes (87%) are ischemic events, caused by blockage of a cerebral blood vessel by a blood clot (2). Occlusion of the cerebrovasculature deprives brain tissue of blood supply, resulting in death of brain cells and development of an area of ischemic infarct. Ischemic stroke can be treated in the acute clinical setting by an endovascular intervention called mechanical thrombectomy, in which the clot is retrieved through the vasculature using an aspiration and/or a stentriever device (3). The so-called “omics techniques” can be extremely useful to advance our understanding of the pathophysiology of ischemic stroke through the study of the thrombotic material extracted by mechanical thrombectomy. Fresh/frozen (FF) specimens are the most used for genomics, proteomics, and metabolomics analysis, but there are practical drawbacks to this approach in a busy clinical setting, reducing availability of FF samples for research. Furthermore, FF samples are expensive to store (4) and to transport to other laboratories for analysis. Formalin-fixation followed by paraffin-embedding (FFPE) is a standard procedure for tissue samples, which allows storage of clinical material at ambient temperature over a long period. Application of mass spectrometry techniques to analyse FFPE tissues is more difficult (5) because the fixation process results in cross-links between proteins and other biomolecules present in the tissue, owing to reactivity of formaldehyde with side chains of amino acids (e.g., lysine, arginine, tyrosine, asparagine, glutamine) (6). These cross-links lead to decreased protein immunoreactivity and extraction from the tissue. Another limitation is the difficulty of paraffin removal (which is water insoluble) without damage or loss of targeted compounds. For these reasons, extraction of biomolecules from FFPE material for analyses using mass spectrometry techniques for proteomics and metabolomics remains a challenging task. Nevertheless, there is great interest in optimizing the use of FFPE tissues in retrospective proteomic studies because they are more practical and versatile compared to FF tissues. Several studies have described different methods of protein extraction from archival clinical samples (7–10).

Using an in-house optimized protein extraction protocol, our study used a shotgun proteomics approach to study the FFPE thrombotic material retrieved from 31 acute ischemic stroke (AIS) patients. To advance understanding of the pathophysiology of AIS, we investigated the proteome of 16 clots of cardioembolic (CE) etiology and 15 clots of large artery atherosclerosis (LAA) etiology.

## Materials and Methods

### AIS Blood Clot Samples

Blood clots were collected from 31 AIS patients as a part of the CÚRAM RESTORE Registry. Samples were collected from four hospitals: Beaumont Hospital, Dublin; Sahlgrenska Hospital, Gothenburg; National Institute of Clinical Neurosciences, Budapest; Metropolitan Hospital, Piraeus. Samples were analyzed by a label-free quantitative liquid chromatography-tandem mass spectrometry (LC-MS/MS) proteomics approach. Prior to this study, approval of the regional hospital ethics committees and National University of Ireland Galway (NUIG) research ethics committees (16-SEPT-08) was obtained in accordance with the ethical standards of the Declaration of Helsinki and its amendments (11). All patients included in the study underwent mechanical thrombectomy between February 2018 and November 2019 for acute occlusion of a large intracranial artery in the anterior or posterior cerebral circulation. The inclusion criteria were: patients >18 years, having undergone mechanical thrombectomy treatment for AIS and having thrombus material available for analysis. Pertinent clinical data were collected from each patient in the form of an anonymized data abstraction form where the etiology of the patient was specified, according to Trial of Org 10172 in Acute Stroke Treatment (TOAST) criteria (12). The TOAST classification system is a widely used subtyping system to classify stroke according to etiology (12). Large artery atherosclerosis and cardioembolism are the two main determined large vessel occlusion stroke subtypes (12). Identifying etiological biomarkers could help in the development of new therapeutics and stroke prevention strategies. Stroke experts performed TOAST classification after complete work-up of the patients in the recruiting hospitals. Thrombi extracted after thrombectomy were collected at the hospital site, stored in pots containing 10% neutral buffered formalin and shipped to the laboratory at NUI Galway for paraffin-embedding and subsequent analysis.

### Sample Preparation Protocol

**Figure 1:**
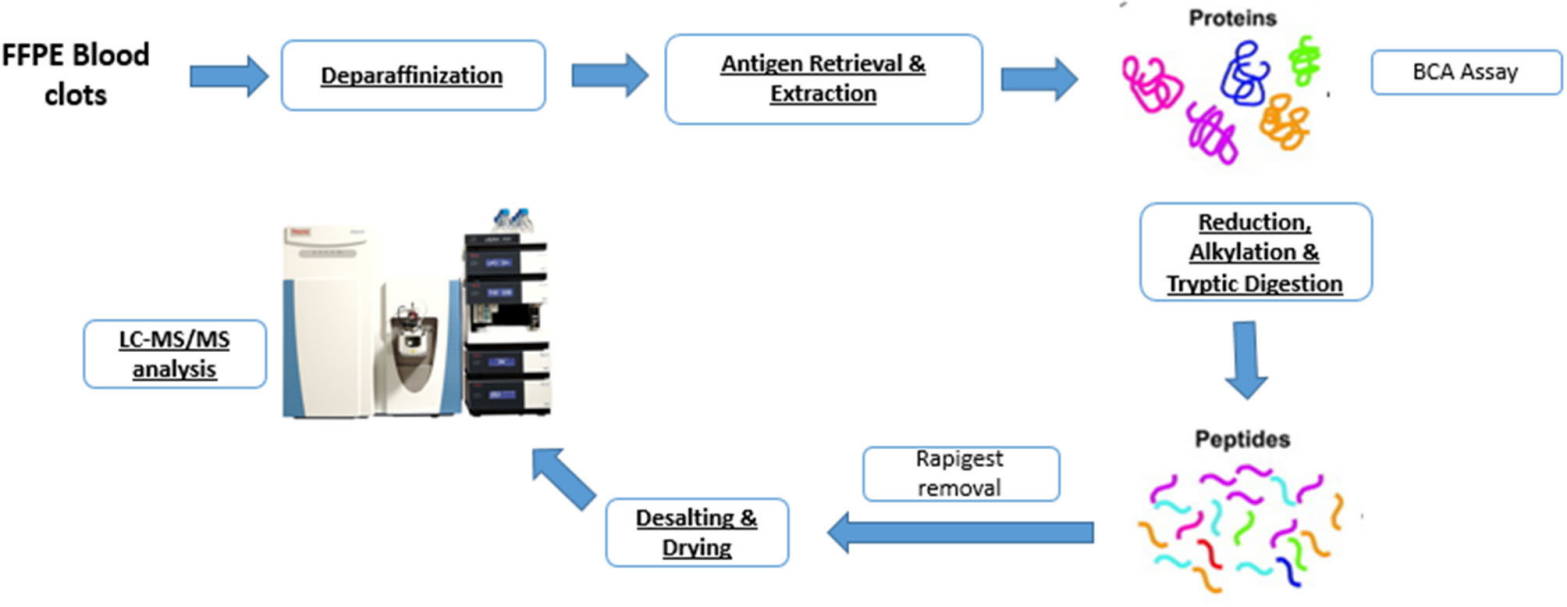
Shotgun proteomics workflow for characterizing the protein content of formalin-fixed paraffin-embedded (FFPE) blood clots.

The sample preparation workflow is schematically represented in Figure 1. Samples were stored in formalin for not longer than 4 weeks to minimize adverse impact of prolonged formalin storage on tissue integrity (13). After formalin fixation and paraffin embedding, approximately 60 μm thick sections (6 sections of 10 μm thickness) were sliced from each FFPE tissue block using a microtome and placed in a 1.5 mL Eppendorf tube. Samples were embedded carefully, maximizing longitudinal distal to proximal orientation, thereby ensuring tissue sections for analysis were representative of the whole clot. Wax blocks were trimmed to expose full-face sections, following which the 6 sections of 10 μm thickness were cut for the analysis.

Our protein extraction protocol followed the method described by Foll et al. (9) with one main adaptation in the deparaffinization/rehydration step. Before protein extraction, deparaffinization was performed by pipetting 1 mL of xylene in the Eppendorf tube containing the sample. After 20 min incubation at room temperature, the tube was then vortexed, centrifuged and the supernatant discarded. The procedure was repeated 2 more times and then, for rehydration, the tissue was incubated twice for 10 min in 100% ethanol, twice for 10 min in 95% ethanol, following 10 min incubation, respectively, with 70 and 50% ethanol. Tissues were then stored in 1 mL water (Liquid Chromatography Mass Spectrometry (LCMS) grade; Sigma-Aldrich, St. Louis, MO, USA) overnight, until further processing.

Extraction was performed by pipetting 200 μL of aqueous buffer containing 0.1% w/v RapiGest (Waters, Milford, MA, USA), 50 mM ammonium bicarbonate (Sigma-Aldrich) pH 8 and 1 mM Dithiothreitol (DTT, Sigma-Aldrich) in each tube containing the sample. The buffered samples were incubated in a thermomixer at 95°C, 750 rpm for 4 h for antigen retrieval and protein extraction. Then, samples were sonicated for 30 min using a waterbath sonicator at room temperature and centrifuged for 20 min at 4°C. The supernatant was collected and the Bicinchoninic acid assay (BCA, Sigma-Aldrich) was used to estimate protein concentration. Samples were then reduced with 3 μL of Tris(2-carboxyethyl)phosphine hydrochloride, (TCEP, Sigma-Aldrich), incubated for 1 h at 37°C and alkylated with 3 μL of 200 mM Iodoacetamide (IAA, Sigma-Aldrich), by incubation for 30 min at room temperature in the dark. Enzymatic digestion was performed by adding 5 μL of 1:1 w/v sequencing grade modified trypsin (Promega, Mybio Ltd) and samples were incubated overnight at 37°C. Trifluoroacetic acid (TFA, Sigma-Aldrich) up to a final concentration of 0.5% v/v (pH <2) was added to stop digestion and precipitate the RapiGest. Samples were then incubated for 1 h at 37°C and centrifuged at 13,000 rpm for 15 min. The supernatant was collected and 5 μg of peptides were desalted by using self-packed C18 zip-tips (Merk Millipore). A speed vacuum concentrator was used to dry the purified peptides, which were then stored at −80°C until shipment to NUI Maynooth for measurement by LC-MS/MS.

### LC-MS/MS Analysis

Vacuum dried samples were dissolved in 20 μL of loading buffer (3% acetonitrile, 97% LC-MS water, and 0.05% trifluoroacetic acid) giving a final concentration of 250 ng/μL. For each run, 4 μL of sample was injected and analyzed by Orbitrap Q-Exactive (Thermo Scientific) coupled to an Easy nano LC 1000 (Thermo Scientific) with 0.3 mL/min flow rate. Peptides were separated by a Thermo RSLC C18 analytical column (2 μm 100A 75 μm 50 cm) using the mobile phases A (97% LC-MS water, 3% LC-MS acetonitrile, 0.1% LC-MS Formic acid) and B (20% LC-MS water, 80% LC-MS acetonitrile, 0.1% LC-MS formic acid). The elution gradient was: 0–5 min 3% [B]; 5–10 min 10% [B]; 10–100 min 40% [B]; 100–105 min 90% [B]; 105–112 min 90% [B]; 112–113 min 5% [B]; 113–133 min 3% [B]. The acquisition was carried out in positive ionization mode (ESI+) and MS was operated in data-dependent mode (Full MS/dd-MS^2^).

### Bioinformatics Analysis

Protein identification and label-free quantification (LFQ) normalization of MS/MS data was performed using MaxQuant v1.5.2.8 (http://www.maxquant.org) (14). Variable modifications selected were Acetyl (Protein N-term) and Oxidation (M), while trypsin was selected as the digestion enzyme and the maximum number of allowed missed cleavage was 2. MS/MS data were correlated against the *Homo sapiens* reference proteome database from Uniprot using the Andromeda search algorithm incorporated in MaxQuant software, including a contaminant sequence set. Data analysis, processing and visualization were performed using Perseus v.1.6.15.0 (www.maxquant.org/) following standard steps for clinical proteomics as described in literature (15). Briefly, normalized peptide intensity values were used to quantify protein abundance for the analysis. Data were filtered to remove protein groups that are only identified by peptides that carry one or more modified amino acids, matching to the reverse database and contaminants. Then log2 transformation was performed, with subsequent grouping of samples according to etiology. Further filtering based on 70% valid values was carried out and a multisample *t*-test was performed. Statistical testing was done at the 2-tailed α level of 0.05 (*p* < 0.05) to identify significantly differentially abundant proteins across the two etiologies. We also used the commonly used permutation based false discovery rate test to correct *p*-values for multiple hypothesis testing. Protein classes were identified using publicly available software programs such as the PANTHER database of protein families (http://pantherdb.org/http://pantherdb.org/) (16) and the STRING database (http://string-db.org) (17) of known and predicted protein interactions that include direct physical and indirect functional protein associations.

## Results

### General Proteomic Profiling of AIS Clots

**Figure 2:**
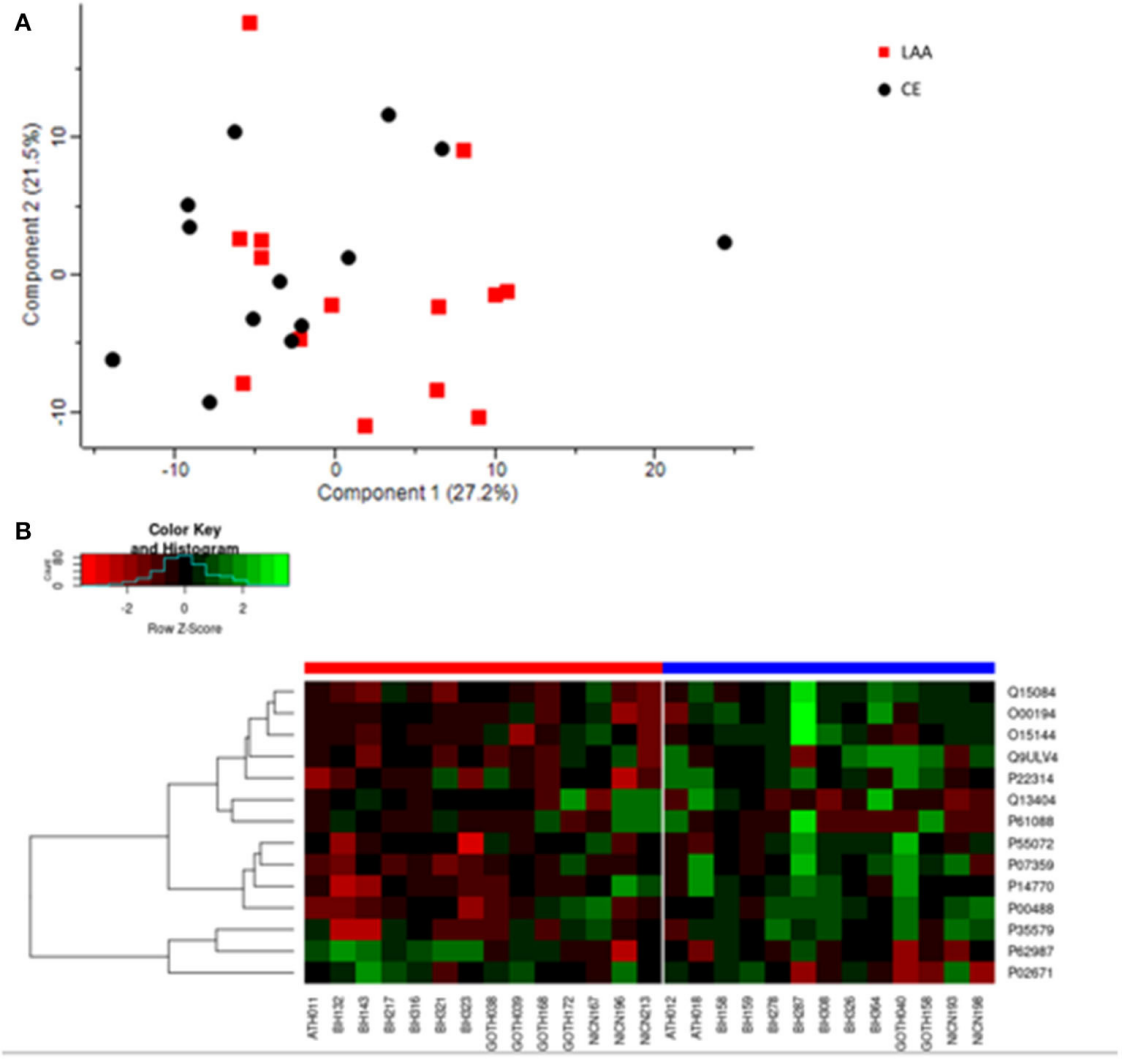
**(A)** Principal component analysis of the general proteome of FFPE AIS clots. LAA clots are indicated with red squares and Cardioembolic (CE) clots are represented by black circles. Each shape indicates individual samples. Component 1 and 2 explain the 27.2 and 21.5% of the sample variation, respectively. **(B)** Heatmap depicting differential expression of the significantly abundant protein groups in LAA (red bar on the left) and CE (blue bar on the right) clots. Protein groups are on the right and indicated with their UniProt ID. In the heatmap, red indicates a decrease in protein abundance, while green indicates an increase in protein abundance.

The general proteome profiling of the analyzed clots was examined to identify differences in protein presence or abundance according to etiology. We identified 1,581 protein groups as part of AIS thrombus proteome (Supplementary Table 1), after removing proteins identified by site, matching to the reverse database and contaminants. After further filtering for at least 70% of valid values (i.e., removing samples and protein groups with <70% valid values), 4 samples were excluded. A total of 179 protein groups (Supplementary Table 2) in the remaining 27 samples (14 LAA and 13 CE) were further analyzed. In 15 cases (8 LAA and 7 CE) recombinant tissue plasminogen activator (rtPA) was administered before mechanical thrombectomy, while in 12 cases (6 LAA and 6 CE) mechanical thrombectomy alone was performed (Supplementary Figure 2). Principal Component Analysis (PCA) was performed based on the protein abundance values from LC-MS/MS to obtain Principal Components (PCs) that explained 48.7% (27.2% PC1 and 21.5% PC2) of data variation among the 27 samples belonging to the two different etiologies (Figure 2A). In Supplementary Figure 1 the PCA has additional labeling identifying the samples by hospital site. No evidence of clustering by collection center is observed.

### Comparative Profiling by Etiology

Adjusted *p*-values did not indicate any significant difference in abundance between CE and LAA clots. However, considering the non-adjusted *p*-values, the comparative proteomic profiling of LAA vs. CE clots resulted in the identification of 14 significantly differentially abundant protein groups between the two different etiologies, four of which were identified as significantly different abundant for LAA stroke etiology (Table 1) and the other ten were identified as significantly different abundant for CE stroke etiology (Table 1). The heatmap in Figure 2B shows the differential expression of the 14 statistically significantly differentially abundant proteins across the 27 samples.

**Table 1:** List of proteins identified as differentially abundant in CE (+) vs. LAA (–) clots.

**Protein name**	**UniProt ID**	**Gene name**	***p***	**Adj *p*^a^**	**Fold change**
Myosin-9	P35579	MYH9	0.012	0,352	+1.47
Coronin-1C	Q9ULV4	CORO1C	0.026	0.537	+1.72
Actin-related protein 2/3 complex subunit 2	O15144	ARPC2	0.032	0.580	+1.30
Platelet glycoprotein Ib alpha chain	P07359	GP1BA	0.023	0.540	+1.61
Platelet glycoprotein IX	P14770	GP9	0.042	0.623	+1.44
Protein disulphide-isomerase A6	Q15084	PDIA6	0.008	0.397	+1.43
Valosin-Containing Protein (VCP)	P55072	VCP	0.049	0.574	+1.48
Ubiquitin-like modifier-activating enzyme 1	P22314	UBA1	0.007	1.000	+1.83
Coagulation factor XIII A chain	P00488	F13A1	0.010	0.392	+1.86
Ras-related protein Rab-27B	O00194	RAB27B	0.021	0.565	+1.36
Ubiquitin-60S ribosomal protein L40	P62987	UBA52	0.048	0.610	−1.68
Ubiquitin-conjugating enzyme E2	P61088	UBE2N	0.007	0.552	−1.44
Ubiquitin-conjugating enzyme E2 variant 1	Q13404	UBE2V1	0.043	0.582	−1.38
Fibrinogen alpha chain	P02671	FGA	0.040	0.656	−1.51

a *Adjusted p-values are calculated according to the permutation based false discovery rate test*.

### Biological Functions and Interaction Patterns of the Significantly Different Abundant Proteins for LAA Stroke Etiology

**Figure 3:**
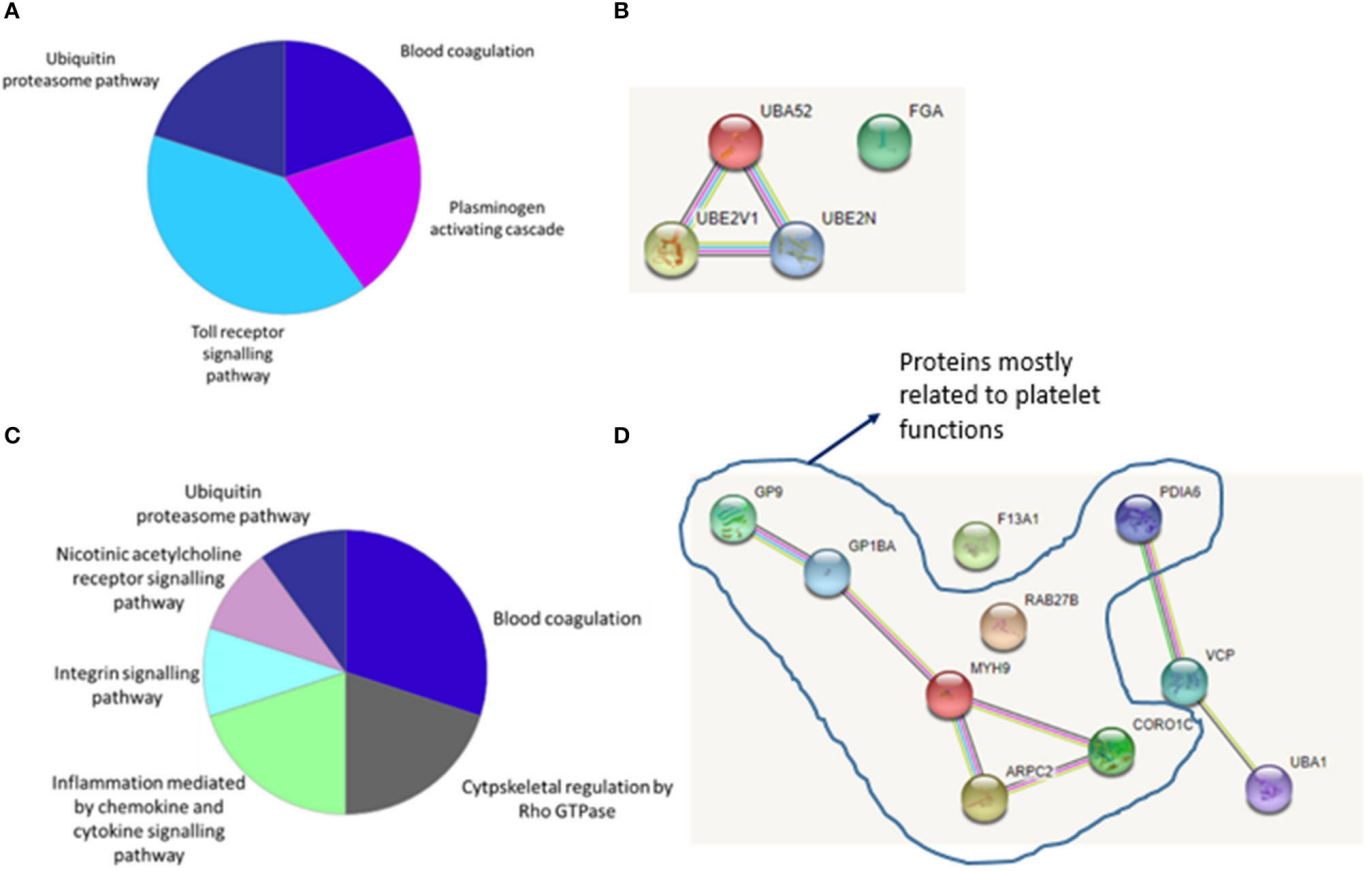
**(A)** Biological pathways and interaction patterns associated with LAA specific proteins. Figure depicts a summary of the biological pathways associated with the specific protein groups identified as having an increased abundance in LAA samples. **(B)** Bioinformatic analysis of the statistically significant proteins using the STRING database identified the interaction patterns with known and predicted protein associations including physical and indirect functional protein linkages. **(C)** Biological pathways and interaction patterns associated with CE specific proteins. Figure depicts a summary of the biological pathways associated with the specific protein groups identified as having an increased abundance in CE samples. **(D)** Bioinformatic analysis of the statistically significant proteins using the STRING database identified the interaction patterns with known and predicted protein associations including physical and indirect functional protein linkages.

Figure 3A shows biological pathways and Figure 3B shows interaction patterns associated with the four significantly different abundant proteins for LAA stroke etiology. The protein groups are involved in four biological pathways: blood coagulation, plasminogen activating cascade, Toll receptor signaling pathway and ubiquitin-proteasome pathway (Figure 3A). Ubiquitin-60S ribosomal protein L40 is part of Ubiquitin-proteasome system (UPS), encoded by the gene UBA52 and its levels increase in response to oxidative stress (18). Its role is closely connected to the proteins Ubiquitin-conjugating enzyme E2, also called Ubc13, and Ubiquitin-conjugating enzyme E2 variant 1 (respectively encoded by genes UBE2N and UBE2V1), which are also significantly more abundant in LAA clots and involved in Toll-like receptor signaling pathway (19) as shown in Figure 3B. The fourth identified significantly different abundant protein, fibrinogen alpha chain (encoded by gene FGA), does not directly interact with the other three protein groups (Figure 3B). The fibrinogen alpha chain is one of the three chains forming the soluble protein fibrinogen and it is involved in both the blood coagulation and plasminogen activation cascade pathways (20).

### Biological Functions and Interaction Patterns of Significantly Different Abundant Proteins for CE Stroke Etiology

Figure 3C shows identified biological pathways and Figure 3D shows interaction patterns for the ten protein groups with significantly different abundant proteins for CE stroke etiology. An increase in proteins related to six biological pathways was observed: blood coagulation, cytoskeletal regulation by Rho GTPase, cytokine and chemokine mediated inflammation, integrin signaling, nicotinic acetylcholine receptor signaling and the ubiquitin-proteasome pathway (Figure 3C). Figure 3D shows the interaction patterns of the significantly different abundant proteins for cardioembolic stroke etiology, with two main clusters, respectively, of five and three protein groups closely interacting and two protein groups not showing any interaction.

One of the main clusters observed consisted of the protein groups coded by the genes GP9, GP1BA, MYH9, ARPC2, and CORO1C. MYH9, ARPC2, and CORO1C are involved in cytoskeletal remodeling of platelets (21–23). GP1BA and GP9 code for proteins that are part of a complex that works as the von Willebrand factor (vWF) receptor and mediates vWF-dependent platelet adhesion to blood vessels, which is a critical initiating event in haemostasis (24).

A second cluster is formed by the protein groups encoded by genes PDIA6, VCP and UBA1, which are associated with the endoplasmic reticulum (ER) and play a role in the ER stress response (25, 26). Finally, although ras-related protein Rab-27B (encoded by gene RAB-27B) and coagulation factor XIII A chain (encoded by gene F13A1) do not show an interaction pattern with the rest of the protein groups, their functions are related to blood coagulation. Ras-related protein Rab-27B is involved in platelet secretion (27) and coagulation factor XIII A chain stabilizes the fibrin mesh in the clot (28).

## Discussion

The rapid development of proteomic techniques in recent years has boosted novel approaches to detect and identify specific proteomes with potential impact in clinical research (29).

In clinical fields, proteomics is used to gain insights into pathological mechanisms and to identify new therapeutic targets by unveiling molecular pathways involved in disease progression. In stroke research, proteomic techniques have the potential to identify biomarkers useful for diagnosis and for secondary stroke prevention. In our study, the primary separation based on sample group in the PCA (component 1) shows some separation of LAA and CE etiologies, which is encouraging. Why for some of the samples there is some level of clustering, while for others there is some overlap is still unclear. It is possible that the presence of other pre-existing conditions, not always diagnosed, which might be common for both CE and LAA etiologies could explain this phenomenon and is worthy of further investigation. Although adjusted *p*-values did not indicate any significant difference in abundance between CE and LAA clots, significant differences in protein abundances between CE and LAA clots were observed in an analysis in which *p*-values were not corrected for multiple hypothesis testing. Adjusted *p*-values are related to sample size, which is quite small in our case. To moderate this limitation, we used the stringent filter of 70% valid values in our analysis workflow producing the non-adjusted *p*-values which show clear statistically significance differences between clots of CE or LAA etiology. Future work with larger sample numbers, with differences in abundance corrected for multiple hypothesis testing is required. However, the present study is encouraging and suggests that FFPE samples analyzed by MS are suitable for biomarker discovery.

Proteomic analysis of FFPE samples is more challenging than using fresh frozen tissues. However, if conditions for proteomic analysis with FFPE samples could be optimized and become routine, this could lead to improved availability of clinical samples for research enabling quicker discovery. Another advantage of using FFPE samples is that tissue could be used e.g., mass spectrometry analysis and histopathologic analysis in tandem.

Many research groups have successfully analyzed the proteome of a various tissue types and disease states using FFPE tissue (7–10), and have shown similarity in the fresh frozen and FFPE proteome (30). We successfully analyzed the proteome of FFPE samples in the current study, identifying 1,581 protein groups as part of the proteome of AIS thrombi using LC-MS/MS. Our results are in line with previous studies performed on FF samples, such as the study of Dargazanli et al. who identified 2,455 proteins in 50 samples (31) and the earlier study by Munoz et al., which used four samples and identified a proteome in the order of 1,600 proteins (32).

Both of these earlier studies found a set of proteins of interest helpful to advance AIS thrombus proteome characterization (31, 32). We acknowledge that our use of FFPE samples allowed us to identify less proteins than previous studies which used FF samples. However, it is encouraging that many of the identified proteins and functional cascades highlighted in this and previous studies using FF tissue are similar. To further advance the feasibility of using FFPE samples in studies such as this, it would be of interest to carry out a direct comparison of the fresh frozen and FFPE proteome of the same sample halved upon collection.

Some of the most significantly enriched pathways found by Munoz et al. in the thrombus proteome included remodeling of epithelial adherents junctions, protein ubiquitination, mitochondrial dysfunction, platelet activation and aggregation and acute inflammatory phase response signaling, including interleukin 6 (IL-6) and Tumor Necrosis Factor alpha (TNF-α) (32). Furthermore, the interactome characterization of the subset of proteins that were common in the four clots, revealed several protein networks that may be involved in thrombus formation, such as the interaction of fibronectin with 14-3-3 proteins. 14-3-3 protein in platelets interacts with several phosphoserine-dependent binding sites in the major platelet adhesion receptor, the glycoprotein Ib-IX complex, regulating its interaction with vWF and initiating thrombus formation (33). Dargazanli et al. compared the proteome of AIS clots of cardioembolic and atheroembolic etiology, and identified seven key proteins that could be indicative of cardioembolic etiology, in particular, coagulation factor XIII and proteins involved in cellular cytoskeletal assembly such as myosin light chain kinase and F-actin capping protein (31). Our results are in line with these findings, since coagulation factor XIII A chain is one of the most abundant proteins we found in CE clots. Additionally, we identified other proteins involved in cytoskeletal activity such as Actin-related protein 2/3 (Arp 2/3) complex subunit 2, Myosin-9, and Coronin-1C.

In our study, we found 14 proteins differentially abundant in clots of the two different etiologies. The protein ubiquitination pathway has been previously related to stroke (34) and our study confirms that several protein groups involved in this pathway are key factors for both CE and LAA etiology. Among the significantly different abundant proteins for LAA stroke etiology there were proteins involved in ubiquitin-proteasome pathway and the coagulation and plasminogen activating cascade. The significantly different abundant proteins for cardioembolic stroke etiology were also related to the ubiquitin proteasome pathway, and the coagulation cascade, but additionally, we identified proteins related to platelet structure and function. Also, a previous study evaluated the proteome of 20 AIS clots from different stroke etiology (35). The study from Rao et al. identified 81 common proteins in all 20 thrombi, while the presence of proteins associated with inflammation (e.g., Integrin Alpha-M) was observed in emboli from patients with high LDL (35). Overall, our findings support the small number of previous studies in the literature demonstrating the potential of mass spectrometry in discriminating thrombi of different stroke etiologies (31, 32, 35). Untargeted proteomics can help researchers to unveil the thrombus proteome, which could provide insights into triggers for thrombus formation, although more studies and targeted analyses are needed. Our findings are in line with our previous findings that CE clots are significantly more platelet and fibrin rich than LAA clots (36). Interestingly, a recent study investigating the proteome of coronary thrombus in patients with ST-segment elevation myocardial infarction, identified five focal adhesion proteins implicated in platelet activation as characteristic of coronary thrombi (37), acknowledging also the important role of platelets in atherosclerotic pathophysiology.

Furthermore, it would be interesting to compare the AIS clot proteome with thrombi retrieved from patients with myocardial infarction and venous thromboembolism, potentially leading to broad and targeted new therapeutic targets.

### Proteins Involved in Ubiquitin-Proteasome System and ER Stress Unfolded Protein Response

Most stroke thrombi are formed in the periphery and subsequently embolise to the brain. The proteins identified reflect the molecular environment of the cells that are part of the thrombus or involved in thrombus development. The results from our study suggest that the ubiquitin-proteasome system (UPS) and ER stress may be part of an inflammatory pathway leading to thrombus formation and subsequently, to stroke. The UPS is a major protein degradation system in eukaryotes (38). A major task of the UPS is to identify and degrade aberrant proteins (misfolded proteins or normal short-lived proteins) which are recognized by the presence of degradation signals, such as small domains or motifs that recruit specific ubiquitin ligases resulting in ubiquitination and degradation of the target protein by the proteasome (38). Although ubiquitin/proteasome-dependent protein degradation is carried out in the nucleus and cytosol, there is a close connection between protein quality control in the endoplasmic reticulum (ER) and the UPS (39). Proteins that fail the ER quality control are transported back to the cytosol where they are rapidly destroyed by the UPS, in a process called Endoplasmic Reticulum Associated Degradation (ERAD) (40). The increase in misfolded/unfolded proteins in the ER lumen triggers the activation of the Unfolded Protein Response (UPR) (41). Our results suggest that both the UPS and the UPR are involved in the molecular mechanisms leading to stroke, since several protein groups identified as most abundant in LAA and CE clots are directly connected with these pathways.

Three out of four protein groups identified as most abundant in LAA clots are part of the UPS, Ubiquitin-60S ribosomal protein L40, Ubiquitin-conjugating enzyme E2 (also called Ubc13), and Ubiquitin-conjugating enzyme E2 variant 1. Ubiquitin-60S ribosomal protein L40, coded by the gene UBA52, is a single ubiquitin fused at the C-terminus to ribosomal protein (RP) L40 (42). It has been shown that the UPS can be directly activated by oxidative stress, both increasing the expression of UBA52 and the ubiquitin-conjugating activity to numerous endogenous substrates (18). The overexpression of UBA52 leads, in turn, to the expression of other antioxidant genes via nuclear translocation of the nuclear factor-like 2 (Nrf2) and to the modulation of P52 toward cell repairing or apoptosis when the stress situation is beyond repair (43). Furthermore, it also influences the activity of FOXO, a transcription factor family whose dysregulation is involved in several cardiovascular diseases (44) and of the pRb pathway, which is a key system of the cell-cycle machinery (45). Interestingly, overexpression of UBA52 was found significantly altered in advanced carotid atherosclerotic plaque in humans (46), which is in line with the findings reported in the present study.

Ubiquitin E2 conjugating enzyme, (Ubc13, and its variant coded by UBE2V1), is another member of the UPS that participates with many different E3 ligases to form lysine 63-linked (Lys63) ubiquitin chains, which are critical to inflammatory signaling and DNA damage response pathways (47). Ubc13 is responsible for non-canonical ubiquitination of TNF receptor-associated factor (TRAF)-family adapter proteins involved in Toll-like receptor and TNF-family cytokine receptor signaling, promoting inflammatory responses (48). It has been suggested that agents reducing Ubc13 activity could have therapeutic utility for treatment of inflammatory and autoimmune disorders (48).

We also found that the significantly different abundant proteins for CE stroke etiology are related to UPS and/or ER stress: Ubiquitin-like modifier-activating enzyme 1 (UBA1), protein disulphide-isomerase A6 (PDIA6) and Valosin-containing protein (VCP).

UBA1 is the E1 enzyme at the apex of ubiquitin signaling in the DNA Damage Response (DDR) (49). This signal transduction pathway that detects lesions in DNA and ensures cell and organismal survival through coordination of DNA repair and DNA replication with physiological processes, including cell cycle progression and transcription (50). Furthermore, UBA1 is also a key component of the UPS, since it catalyzes the first step in ubiquitin conjugation to mark cellular proteins for degradation via the proteasome (51) and has been implicated in the development of cancer and neurodegenerative diseases (51, 52).

PDIA6 is a protein disulphide isomerase present in the eukaryotic ER (53) that negatively regulates the UPR following ER stress (25). An *in-vivo* study in mice showed that PDIA6 can protect cardiac myocytes against simulated ischemia/reperfusion-induced death in a manner that is dependent on the catalytic activity of the enzyme (54). Furthermore, PDIA6 plays a role in a number of pathways related to platelet activities, such as platelet aggregation, secretion and fibrinogen binding (55).

VCP activity is required for maintenance of normal ER structure and function (26) and it helps in the extraction of terminally misfolded polypeptides from the ER into the cytosol for degradation by the UPS in the ERAD process (56).

It has also been reported that ER stress and the activation of UPR might be a driving force behind the formation of proplatelets that generate platelets (57). A recent study observed that megakaryocyte differentiation is associated with caspase activation and can be inducted by ER stress (58).

### Proteins Involved in Blood Coagulation

Fibrinogen alpha chain is one of the three chains forming the soluble protein fibrinogen and one of the most abundant proteins in LAA clots. Apart from its crucial role in coagulation (20), several studies highlight that the C-terminal domain of the fibrinogen α chain (αC domain) is implicated in different clotting abnormalities (59), amyloid deposits in Alzheimer's disease (60) and familiar renal amyloidosis (61). For example, there is some evidence that the α-fibrinogen T312A variant might influence clot structure through increased factor XIII cross-linking, leading to the formation of fibrin clots that could predispose to embolization (62, 63). Furthermore, it seems that the fibrinogen associated with Aβ peptides might contribute to the development of Alzheimer's disease by accumulating around or inside brain blood vessels (64), provoking the degeneration of vessel wall components, adversely affecting cerebral blood flow and worsening cognitive decline (65).

Seven of the significantly different abundant proteins for CE stroke etiology are related to platelet function.

The proteins myosin-9, coronin-1C and actin-related protein (Arp) 2/3 complex subunit 2, for example, are in involved cytoskeletal remodeling of platelets (21–23), which undergo profound shape changes during activation and adhesion (66). Myosin-9 plays a crucial role in organelle distribution and F-actin organization in megakaryocytes and platelets (21) and mutations in the MYH9 gene have been related to several coagulation disorders (67). A recent case report described a correlation between MYH9-related thrombocytopenia and Moyamoya-like vasculopathy, a disorder defined by progressive occlusion or stenosis of the intracranial distal segments of the internal carotid arteries and the arteries of the circle of Willis, provoking ischemic stroke events (68). Coronin-1C works as a regulator of the actin cytoskeleton turnover (69) together with actin-related protein 2/3 complex (23, 70). There are several studies indicating the relevance of members of the evolutionarily conserved coronin family of proteins in stroke and thrombus formation, since they are crucial for platelet function (23), but also for controlling lipoprotein uptake and degradation in macrophages (71). Furthermore, coronins, specifically overexpression of coronin-1C, has been implicated in several cancers (72–75), therefore, it may be an interesting starting point for the study of stroke-related tumors.

The platelet glycoproteins Ib alpha chain (GPIbα) and IX are two other protein groups found significantly different abundant for CE stroke etiology. Their role is extremely interconnected since they are part of the glycoprotein Ib-IX-V complex (24), which is a crucial platelet receptor for initial tethering and adhesion at sites of vascular injury. In particular, upon vascular damage, the vWF protein is exposed and binds to GPIbα, which is responsible for the initial platelet adhesion, as shown by the bleeding disorders Bernard Soulier syndrome (76) and von Willebrand disease (77). Studies in mice demonstrated that GPIbα is an important mediator of ischemic stroke, suggesting that targeted inhibition of this receptor may open new approaches for stroke treatment (78), especially in view of the thromboinflammatory axis played by VWF-GPIbα interaction in acute ischemic stroke (79).

The coagulation factor XIII A chain is part of coagulation factor XIII, a heterotetrameric protein complex consisting of a dimer of A subunits (FXIIIA_2_), and a dimer of B subunits (FXIIIB_2_) (80, 81). The role of this protein in blood clotting is extremely important since its activation, by thrombin and Ca^2+^ represents the last step of the coagulation cascade (82). The FXIIIA_2_ subunit is the catalytic subunit and it has transglutaminase activity that covalently cross-links fibrin polymers to confer stability to the blood clot and resistance against premature fibrinolysis (83). Apart from its physiological activity, there are several studies that showed out how polymorphisms in coagulation factor XIII (especially related to the A subunit) might be linked to thrombotic diseases (84–86) and possibly to an increased risk of acute ischemic stroke, especially in patients with coronary artery disease and/or myocardial infarction (86, 87). These findings are in line with our results and suggest that coagulation factor XIII A should be further investigated as a possible biomarker for AIS, possibly related to cardioembolic etiology.

Finally, Ras-related protein Rab-27b (encoded by the gene RAB27B) is another protein we found as significantly different abundant proteins for CE stroke etiology. Like the majority of the protein groups most abundant in CE clots, its function is related to platelets, as highlighted by a previous study investigating the platelet proteome (88). There is evidence that this protein is a key regulator of dense granule secretion in platelets (27). In addition, ras-related protein Rab-27b belongs to the class of the Rab proteins, which are gaining recognition as important in the biogenesis and membrane structure of exosomes and may have therapeutic and diagnostic potential for a number of pathologies, included cancer, stroke and neurological disorders (89, 90).

### Study Strengths and Limitations

As previously mentioned, extraction of biomolecules from FFPE material for mass spectrometry analysis remains a challenging task. However, we obtained a number of proteins comparable to FF samples used in previous studies present in literature, which is the major strength of our study. We acknowledge some limitations, such as the relatively small number of samples we included in our study. We also acknowledge that pre-stroke anticoagulant therapies and the treatment with recombinant tissue plasminogen activator before mechanical thrombectomy might also modify the proteome of the thrombus. However, as in our cohort, the percentages of rtPA yes and rtPA no patients are similar across the two etiologies, this makes it unlikely that thrombolysis is a confounder in our study. However, it would be valuable to probe in more depth the impact of potential confounders such as thrombolysis, prior medication, age, sex, comorbidities and stroke risk factors on the thrombus proteome in future studies.

## Conclusion

This proof-of-principle study demonstrates the feasibility of mass spectrometry to characterize the proteome of FFPE blood clots as well as the possibility to identify specific proteins that may differentiate between CE and LAA etiology. Further studies with larger cohorts of patients are needed. Our results confirm that quantitative proteomic studies performed on the clots that cause stroke in AIS patients can unveil important information to enrich our understanding of stroke pathophysiology.

## Data Availability Statement

The datasets presented in this study can be found in online repositories. The name of the repository and accession number can be found below: Mass Spectrometry Interactive Virtual Environment (MassIVE), https://massive.ucsd.edu/ProteoSAFe/static/massive.jsp, MSV000088792'.

## Ethics Statement

The studies involving human participants were reviewed and approved by prior to this study, approval of the Regional Hospital Ethics Committees and National University of Ireland Galway (NUIG) Research Ethics Committees (16-SEPT-08) was obtained in accordance with the ethical standards of the Declaration of Helsinki and its amendments. The patients/participants provided their written informed consent to participate in this study.

## Author Contributions

KD obtained the funds for the study, coordinated the implementation of the study, and supervised the writing of the manuscript. RR participated in samples collection, developed the study design, developed the proteome extraction method, performed the bioinformatic analysis, composed the manuscript, and wrote the results and discussion. OM participated to the development of the extraction protocol and helped in sample collection. MB, SO'C, and CT participated in performing the bioinformatic analysis. SM and AD participated in samples collection, analysis, and interpretation of results. KP, GT, AR, and JT performed the thrombectomy at the several hospitals, evaluated stroke etiology, and collected samples and procedural data. KP, GT, IS, TT, AR, and JT contributed to the study design and were responsible thrombus collection at the relevant stroke center. AP, MG, and RM contributed to develop the study design and funding acquisition. PÓ supervised the bioinformatic analysis. All authors have read and reviewed the manuscript.

## Funding

This publication has emanated from research conducted with the financial support of Science Foundation Ireland (SFI) and is co-funded under the European Regional Development Fund under Grant No. 13/RC/2073_2. Furthermore, the authors declare that this study received funding from Cerenovus. The funder was not involved in the study design, collection, analysis, interpretation of data, the writing of this article or the decision to submit it for publication.

## Conflict of Interest

MG and RM were employed by company Cerenovus. The remaining authors declare that the research was conducted in the absence of any commercial or financial relationships that could be construed as a potential conflict of interest.

## Publisher's Note

All claims expressed in this article are solely those of the authors and do not necessarily represent those of their affiliated organizations, or those of the publisher, the editors and the reviewers. Any product that may be evaluated in this article, or claim that may be made by its manufacturer, is not guaranteed or endorsed by the publisher.
