# Supplementary figures and images for: Potential Biomarkers of Acute Ischemic Stroke Etiology Revealed by Mass Spectrometry-Based Proteomic Characterization of Formalin-Fixed Paraffin-Embedded Blood Clots
Source: Front Neurol. 2022 Apr 19;13:854846. doi: 10.3389/fneur.2022.854846 (PMC9062453; doi:10.3389/fneur.2022.854846)

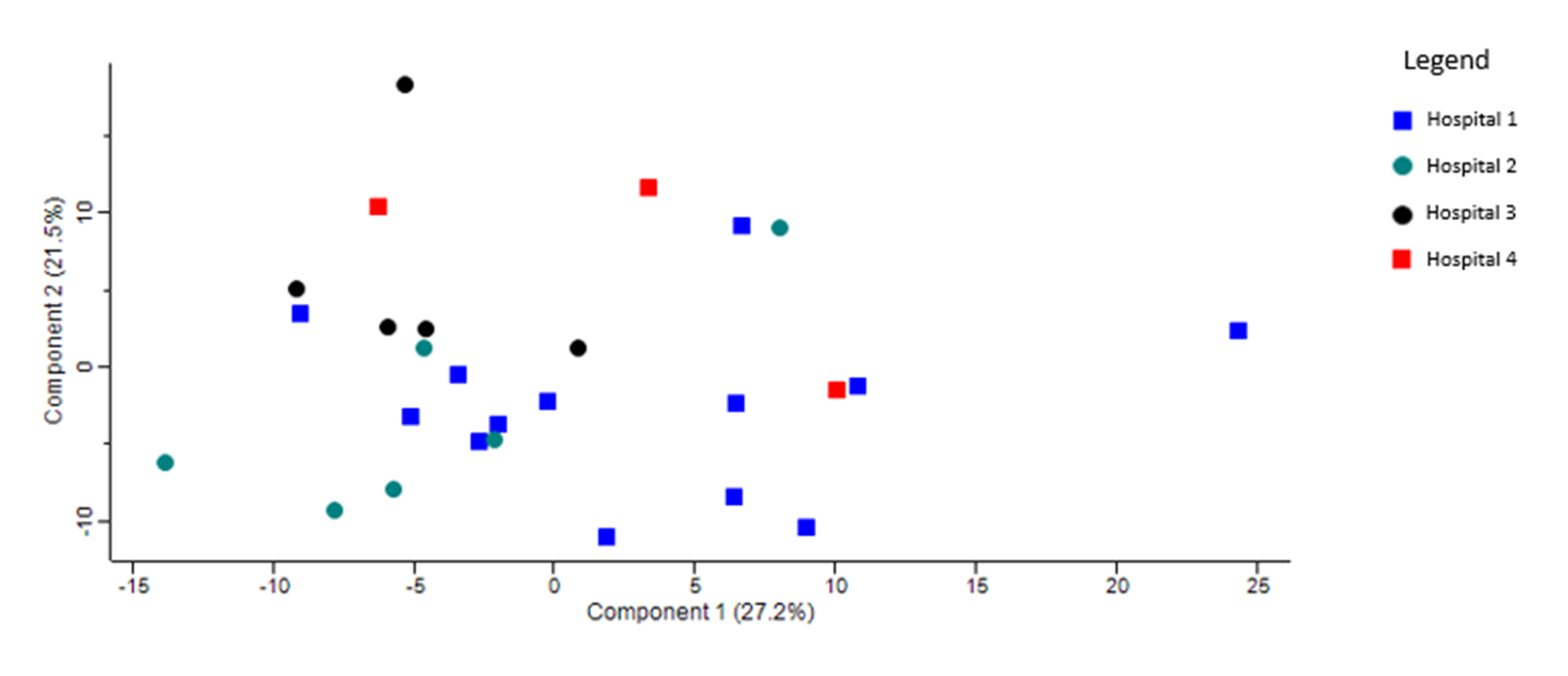

Supplement: Supplementary file 1 [file Image_1.TIFF]

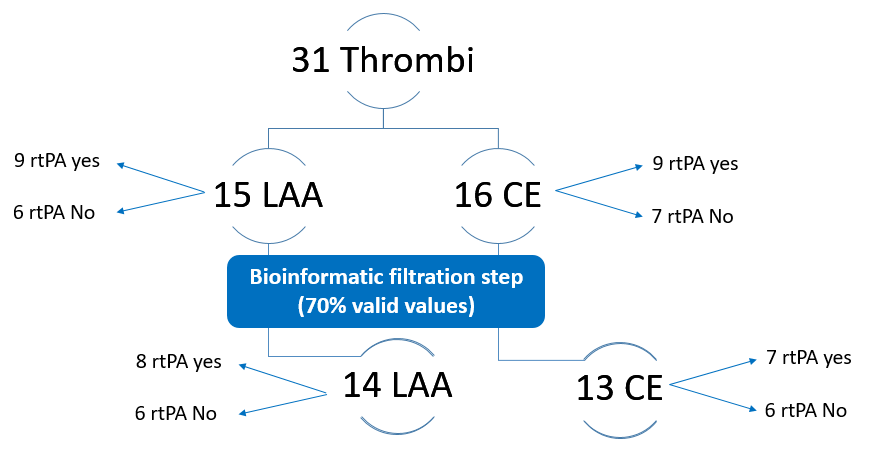

Supplement: Supplementary file 2 [file Image_2.TIFF]
